# New Diagnostic and Therapeutic Approaches in Otologic Diseases

**DOI:** 10.1155/2013/610748

**Published:** 2013-12-10

**Authors:** Fatih Oghan, Cemal Cingi, Erkan Karatas, Sebahattin Cureoglu

**Affiliations:** ^1^Department of Otolaryngology Head and Neck Surgery, Evliya Celebi Training and Research Hospital, Dumlupinar University, 43270 Kutahya, Turkey; ^2^Department of Otolaryngology Head and Neck Surgery, Faculty of Medicine, Osmangazi University, Meselik Campus, 26480 Eskisehir, Turkey; ^3^Department of Otolaryngology Head and Neck Surgery, Turgut Ozal Medical Center, Inonu University, 44280 Malatya, Turkey; ^4^Department of Otolaryngology, University of Minnesota, MN 55369, USA

Cochlear pathologies and vestibular disorders have been investigated in detail in the recent years. Recent advances in diagnostic and therapeutic methods have introduced new investigations about the cytologic degenerations and regenerations of cochlear cells. Knowledge and understanding of otologic diseases have led to the development of novel therapies, approaches, and/or tools.

This special issue covers the important topics in the diagnosis and therapies of otologic diseases including chronic otitis media, tinnitus, ototoxicity, and hearing loss.

*Investigation of the Presence of Biofilms in Chronic Suppurative Otitis Media, Nonsuppurative Otitis Media, and Chronic Otitis Media with Cholesteatoma by Scanning Electron Microscopy.* Biofilms have been shown to play a major role in the pathogenesis of otolaryngologic infections. However, very limited studies have been undertaken to demonstrate the presence of biofilms in tissues from patients with chronic otitis media (COM) with or without cholesteatoma. E. Kaya et al. found that their study supports the hypothesis that biofilms are involved in chronic suppurative otitis media, cholesteatoma, and to a lesser degree, chronic nonsuppurative otitis media. The presence of biofilms was significantly higher in the middle ear mucosa compared with the mastoid and ossicle samples. They emphasized that careful use of topical or systemic antimicrobials is essential and during surgery, hypertrophic tissue must be carefully removed from normal tissue. 

*Is It Necessary to Do Temporal Bone Computed Tomography of the Internal Auditory Canal in Tinnitus with Normal Hearing?* Tinnitus is the perception of sound without an external stimulus. The prevalence of tinnitus varies between 3–30% of all population. As we know very well, tinnitus can be seen due to the pressure of the acoustic neuromas, cerebellopontine angle tumors, and vascular lesions, such as vascular loop to the eight cranial nerve reported in the literature. The development of the tinnitus can be observed due to nerve edema, degeneration, and compression in the canal. Accordingly, the pathological conditions that affect the width of the canal can lead to tinnitus due to compression. T. L. Kumral et al. investigated the diameter of internal acoustic canal in physiologically impaired tinnitus patients as the etiology may be due to anatomical differences of the temporal bone. In conclusion they found that there were no anatomical differences in the etiology of tinnitus rather than physiological degeneration in the nerves.

*Efficacy of Low-Level Laser Therapy in the Management of Tinnitus due to Noise-Induced Hearing Loss: A Double-Blind Randomized Clinical.* Several remedial modalities for the treatment of tinnitus have been proposed, but an effective standard treatment is still to be confirmed. In the present study, A. Mollasadeghi et al. investigate the effect of low-level laser therapy on tinnitus accompanied by noise-induced hearing loss. They found low-level laser therapy to be effective in alleviating tinnitus in patients with noise-induced hearing loss, although this effect has faded after 3 months of follow-up.

*The Role of Thiamine Pyrophosphate in Prevention of Cisplatin Ototoxicity in an Animal Model*. Cisplatin ototoxicity has several characteristics. Inner ear toxicity is often a dose limiting side effect that hampers optimal cisplatin-based chemotherapy. It is normally manifested as a sensorineural hearing loss beginning in the high frequencies, successively progressing towards the speech frequency range. O. Kuduban et al. investigate the effectiveness of thiamine pyrophosphate against cisplatin-induced ototoxicity in guinea pigs. They found that systemic administration of thiamine pyrophosphate yielded statistically significant protection to the cochlea of guinea pigs from cisplatin toxicity. They emphasized that further experimental animal studies are essential to determine the appropriate indications and dosages of thiamine pyrophosphate before clinical use.

*Extended High Frequency Audiometry in Polycystic Ovary Syndrome*. Polycystic ovarian syndrome (PCOS) is the most common endocrine disorder affecting 5–10% of women in the reproductive age. Disease is characterized by oligo/amenorrhea, hyperandrogenism, and polycystic ovaries. PCOS is a chronic proinflammatory state. C. Kucur et al. evaluated extended high frequency hearing loss in PCOS patients. They concluded that PCOS patients have hearing impairment especially in extended high frequencies. Further studies are needed to help elucidate the mechanism behind hearing impairment in association with PCOS and to see whether the impairment of extended high frequency audiometry in these cases is progressive.

*Possible Protective Effect of Sertraline against Cisplatin-Induced Ototoxicity: An Experimental Study.* Cisplatin is a widely used chemotherapeutic agent but its ototoxicity side effect can occur in the majority of patients. Lots of agents were tried to prevent this, but there is not a routine treatment modality yet. The aim of this study was to evaluate the otoprotective effect of sertraline, which is an antidepressant with neuroprotective effects, against cisplatin, in rats. M. Ozturk et al. showed that sertraline seems to have a protective effect on cisplatin ototoxicity, and could be used to prevent the ototoxicity and also to treat the depression that occurred in cancer patients together.

*Fatih Oghan* *Fatih Oghan* *Cemal Cingi* *Cemal Cingi* *Erkan Karatas* *Erkan Karatas* *Sebahattin Cureoglu* *Sebahattin Cureoglu*

